# Changes in the Fitness Fatness Index following reduced exertion high-intensity interval training versus moderate-intensity continuous training in physically inactive adults

**DOI:** 10.3389/fspor.2022.961957

**Published:** 2022-08-05

**Authors:** Daniel J. Leahy, Lance C. Dalleck, Joyce S. Ramos

**Affiliations:** ^1^Caring Futures Institute, SHAPE Research Centre, Clinical Exercise Physiology, College of Nursing and Health Sciences, Flinders University, Adelaide, SA, Australia; ^2^Recreation, Exercise and Sport, Western Colorado University, Gunnison, CO, United States

**Keywords:** cardiorespiratory fitness, body composition, interval training, reduced exertion high-intensity interval training, fitness fatness index

## Abstract

**Background:**

Many adults do not reach the recommended exercise participation guidelines, often citing lack of time as a barrier. Reduced exertion high-intensity training (REHIT) is a mode of exercise that takes as few as 10 min and has been shown to be as effective as other modalities. The Fitness Fatness Index (FFI) is a recently developed index that is used to predict cardiovascular disease (CVD) risk. The aim of this study was to determine the efficacy of a REHIT vs. a traditional moderate-intensity continuous training (MICT) on FFI in physically inactive adults.

**Methods:**

Thirty-two participants were randomized into one of two 8-week exercise intervention groups: (i) REHIT (*n* = 16); (ii) MICT (*n* = 16). The REHIT group performed 10 min of individualized cycling intervals on 2–4 days of the week. The MICT group were prescribed aerobic exercise at 50–65% of their heart rate reserve (HRR) on 3–5 days of the week. FFI was recorded at baseline and post 8-weeks, with FFI being calculated as cardiorespiratory fitness (CRF) (expressed as metabolic equivalents) divided by waist to height ratio (WtHR). A 1-unit increase in FFI was recognized as a clinically significant change in FFI.

**Results:**

The REHIT group showed significantly greater (+1.95, ±0.63) improvements in FFI compared to those in the MICT (+0.99, ±0.47) group (between group difference, *p* < 0.001). Furthermore, there was a greater proportion of participants who achieved a clinically significant change in FFI in the REHIT group (12/12, 100%) than in the MICT group (8/15, 53%) (between group difference, *p* = 0.01).

**Conclusion:**

This study suggests that REHIT may be a more efficacious exercise modality to increase FFI than MICT. This outcome is beneficial as the clinician can prescribe REHIT to physically inactive adults who cite lack of time as a barrier to physical activity participation and achieve significant reductions in CVD risk.

## Introduction

Physical inactivity is commonly associated with an increased risk of suffering from cardiovascular disease (CVD) and its associated risk factors including reduced quality of life, and poor mental health (Hussain et al., 2016). Despite the adverse impacts of physical inactivity, many adults do not reach the recommended physical activity guidelines of 150–300 min of moderate-intensity or 75–150 min of vigorous intensity per week (Hoare et al., 2017). Lack of time is commonly reported as a barrier to participation in physical activity (Hoare et al., 2017). Moderate-intensity continuous training (MICT) is recognized as the traditional form of aerobic exercise and is recommended for reducing an individual's risk of physical inactivity-related diseases (Garber et al., 2011). This method, however, is more time consuming than alternative exercise modalities (Metcalfe et al., 2012). Reduced-exertion high-intensity interval training (REHIT) is one of such modalities, a version of sprint interval training (SIT) consisting of short durations and low repetitions of high-intensity exercise, lasting for no longer than 10 min per bout (Metcalfe et al., 2012). REHIT however is still able to elicit great cardiometabolic benefits in healthy sedentary adults (Metcalfe et al., 2012). This allows for REHIT to potentially be a time efficient yet effective exercise method to increase participation and reduce the risk of disease related to physical inactivity. Previous research has indicated that a similar volume of interval training is more effective than MICT at improving both fitness and fatness in adults with metabolic syndrome (Ramos et al., 2017).

The fitness fatness index (FFI) is an index which was developed to better detect the joint contributions of fitness and fatness in healthy and at risk individuals with health outcomes such as incident diabetes and all-cause and CVD-specific mortality risk (Sloan et al., 2016). The individual's cardiorespiratory fitness (CRF), either measured or predicted, and expressed as the metabolic equivalent (MET), is divided by waist to height ratio (WtHR) to develop an index (Sloan et al., 2016). This index has been shown to be a greater predictor of CVD, when compared to using either fitness or fatness individually (Edwards et al., 2017). FFI improvement could therefore be an important target in improving overall cardiovascular health, with a one unit increase in FFI shown to be associated with a 9 and 11%, reduction in all-cause and CVD-specific mortality, respectively (Frith and Loprinzi, 2017). This makes the FFI a beneficial outcome measure for general practice as the clinician can use two easily obtainable measures to better track changes over the course of an exercise intervention and predict an individual's risk of CVD. Despite this, no previous research has used the FFI as an outcome measure to evaluate the efficacy of an individualized REHIT exercise intervention.

This study aimed to determine whether REHIT is more effective than traditional MICT in improving the FFI in physically inactive individuals. It was hypothesized that REHIT would be a more efficacious exercise modality than MICT to increase FFI in this cohort.

## Methodology

Participants included 32 adults (male, *n* = 16) who were staff at a local university and hospital. Recruitment occurred through an advertisement on a university website, a local newspaper, and word of mouth. Eligible participants included those who were low to moderate risk to perform exercise and were classified as physically inactive according to the American College of Sports Medicine classification (Pescatello, 2014). Participants were excluded from the research if they showed any evidence of metabolic, cardiovascular, or pulmonary disease. Approval for this study was granted by the Human Research Committee at Western State Colorado University. Those who were eligible to be included in the study signed an informed consent form prior to commencing the research. Sample size was calculated based on CRF change as the main outcome variable. Our sample size calculation revealed that we need 16 participants in each study group to detect a 1.0 SD difference between groups with 80% power (Skinner et al., 2001).

### Chronic effect of REHIT vs. MICT on FFI

**Figure 1:**
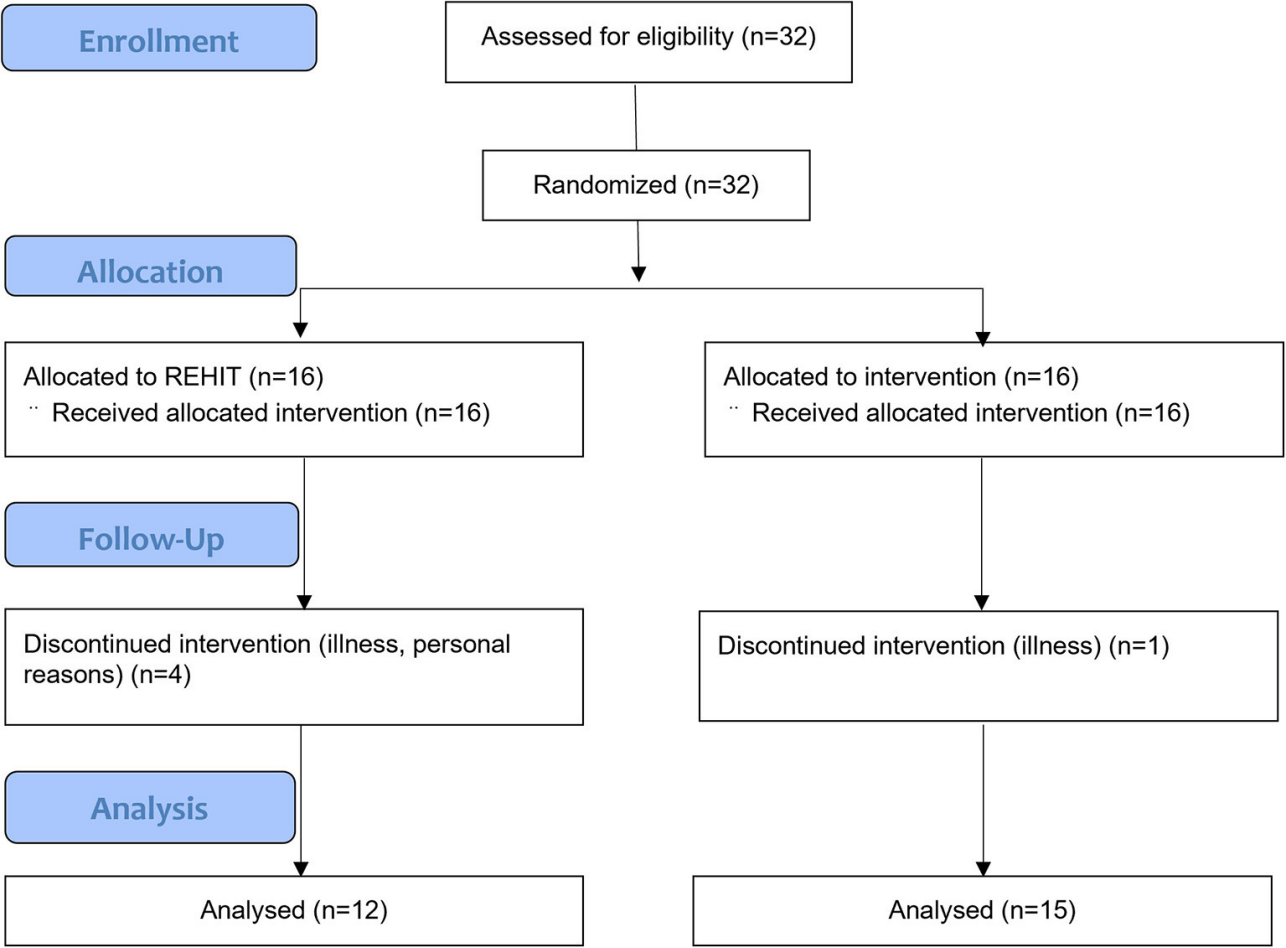
Consort flow diagram for FFI study.

Figure 1 displays the process of group allocation and exercise intervention following recruitment. After recruitment, participants were randomized to one of the exercise intervention groups. Participants performed baseline testing before completing 8 weeks of supervised exercise according to their group allocation.

- Group one (*n* = 16) were prescribed 8 weeks of a REHIT program performed on a CAR.O.L exercise bike. The high intense sessions consisted of a 3-min warm up, followed by a 20 s sprint, 3-min recovery, 20 s sprint, and 3-min cool down. The REHIT sessions were performed at the participants workplace (university and hospital).
- Group two (*n* = 16) were prescribed an 8-week MICT program following the standardized aerobic exercise prescription of 30 min of aerobic exercise performed at 50–65% heart rate reserve (HRR), on 5 days of the week.

Participants performed a maximal exercise test at baseline and post the 8-week intervention. This included a graded test on a cycle ergometer, determining the participant's maximal cardiorespiratory fitness and maximal heart rate. Anthropometric data was collected at baseline, and 48–72 h post the final session. These measures collectively formed the FFI, which was then used to compare the efficacy of an 8-week intervention of REHIT vs. MICT.

The difference between resting and maximum heart rate values from the baseline exercise test was calculated for HRR. For the MICT exercise sessions, percentage of HRR was identified by converting the required percentage of HRR to a decimal by dividing by 100, multiplying this number by the individual's HRR, and adding this value to their resting heart rate.

### REHIT exercise sessions

The REHIT group performed their prescribed exercise on a CAR.O.L cycle ergometers, a specially designed exercise cycle by Integrated Health Partners (London, UK). The use of this cycle allows the REHIT sessions to be individualized, as the CAR.O.L cycle ergometer adjusts the resistance of the ergometer progressively as the participant becomes fitter. This is done by taking into account the weight, power output and fatigue index of the participants using a built-in self-learning algorithm, and adjusting the resistance accordingly. The specific intensities, durations and frequencies for the exercise sessions of the REHIT group are as follows:

*Week 1:* High-intensity cycle intervals, 2 days per week, 10 min per session.

*Week 2:* High-intensity cycle intervals, 2 days per week, 10 min per session.

*Week 3:* High-intensity cycle intervals, 3 days per week, 10 min per session.

*Week 4:* High-intensity cycle intervals, 3 days per week, 10 min per session.

*Weeks 5-8:* High-intensity cycle intervals, 4 days per week, 10 min per session.

### MICT exercise sessions

In accordance with the guidelines published by the American College of Sports Medicine (Pescatello, 2014), participants were prescribed the standardized moderate-intensity aerobic exercise, aimed to reach a volume of 150 min over 5 days of the week. Given the participants were previously inactive, the volume and intensity of exercise started below the guideline and gradually progressed to the standardized recommended levels. The progression of intensities, durations and frequencies of the exercise prescription are outlined below:

*Week 1:* 40-50% of HRR, 3 days per week, 25 min per session.

*Week 2:* 50-55% of HRR, 4 days per week, 30 min per session.

*Weeks 3-4:* 55-60% of HRR, 4 days per week, 30 min per session.

*Weeks 5-6:* 55-60% of HRR, 5 days per week, 30 min per session.

*Weeks 7-8:* 60-65%% of HRR, 5 days per week, 30 min per session.

### Anthropometric measurements

A medical grade scale stadiometer (Tanita Corporation WB-3000, Tokyo, Japan) was used to measure the participants' height to the nearest 0.5 cm. Participants had their waist circumference measured using a cloth tape measure with a spring loaded-handle, with the measurement taken at the narrowest point between the xiphoid process and umbilicus and recorded in cm. Waist measurements were taken until two measures were within 0.5 mm.

### Maximal graded exercise test

Participants performed a graded maximal exercise test on a cycle ergometer with heart rate and gas exchange values being recorded. A warm up consisted of 2 min of pedaling at 50 Watts. During the test, a step protocol was performed, with the power output increasing until volitional fatigue after 7–11 min. This included an increase of five watts every 20 s for men, and five watts every 30 s for women, with revolutions per minute remaining at 70–90 throughout the test. Volitional fatigue was considered obtained when the participant could not sustain a cadence above 40 revolutions per minute. To determine whether the test results could be considered a maximal measure of CRF, two out of the three following criteria needed to be evident: (1) a plateau in VO_2_ (≤ 150 mL/min) despite an increase in workload, (2) a respiratory exchange ratio ≥1.1, and (3) the participant's maximal heart rate being within 15 beats per minute of the age predicted maximal heart rate (220-age).

### Fitness fatness index

FFI was calculated by dividing CRF by WtHR. Participant's maximal VO_2_ recording was converted to METs (VO_2_/3.5), while WtHR was determined by dividing the participant's recorded waist circumference in cm by their recorded height in cm. These two values (CRF in METs and WtHR) were then divided to obtain the FFI. FFI scores usually range between 10 and 50 on a continuous scale, with higher scores being better (Sloan et al., 2016).

### Statistical analysis

All data analysis were performed using SPSS version 27. The average and spread of the data included are presented as mean ± standard deviation (SD). A Shapiro-Wilk test was initially performed to determine the normality of the data. A two-way ANCOVA test was used to determine the difference in FFI change from pre- to post-intervention between training groups. The baseline FFI was used as the covariate, while the FFI at post 8 weeks of the given exercise intervention was the dependent variable in the analysis. A Chi-Square test was also performed to identify the difference in proportion of likely responders to a clinically significant FFI change between exercise groups. Participants were classified as a likely responder if they showed an increase in FFI of ≥1 unit, the value representing a clinically significant reduction in CVD risk (Frith and Loprinzi, 2017). Statistical significance was set at *p* < 0.05 for all analyses.

## Results

Data analyses were performed on all participants who completed 8 weeks of their allocated intervention. There were four participants in the REHIT group who did not complete the study due to illness (*n* = 3) and personal reasons (*n* = 1), while 1 participant did not complete the MICT intervention due to illness. The REHIT and MICT groups did not differ significantly in baseline measures. Mean adherence to exercise in the REHIT group was 89.2% (range, 75–100%), while mean adherence in the MICT group was 87.8% (range, 77.1–100%). Participant characteristics are shown in Table 1. The outcome measures of the participants at baseline and post 8 weeks of intervention are displayed in Table 2.

**Table 1:** Participant characteristics.

**Variable**	**REHIT**	**MICT**
Participants (males)	12 (5)	15 (6)
Age (years)	40.8 (10.8)*	42.9 (9.7)*
Height (cm)	172.8 (9.8)*	170.1 (10.9)*

* Values displayed are mean (±SD).

**Table 2:** Outcome measures of participants at baseline and post 8 weeks of intervention.

**Variable**	**REHIT (*****n =*** **12)**		**MICT (*****n =*** **15)**	
	**Baseline**	**8 weeks**	**Baseline**	**8 weeks**
Waist circumference (cm)	86.6 (10.5)	85.4 (9.7)	87.6 (10.1)	87.3 (9.1)
Waist to height ratio	0.50 (0.06)	0.49 (0.06)	0.51 (0.05)	0.51 (0.05)
VO_2_max (mL/kg/min)	25.3 (2.9)	28.4 (3.1)	26.2 (6.7)	28.0 (7.1)
METs	7.24 (0.82)	8.12 (0.88)	7.48 (1.92)	7.99 (2.03)
FFI	14.65 (2.72)	16.60 (2.59)*#	14.84 (4.75)	15.82 (4.80)*

Values displayed are mean (±SD); ^*^Significantly greater than baseline measure within-group (p < 0.05); #Change from baseline significantly greater than in MICT group (p < 0.05).

### Changes in FFI

Table 2 shows the efficacy of the 8-week exercise interventions on the main outcome measure, FFI. The mean increase of FFI in the REHIT group was 1.95 ± 0.63 units, while the MICT group showed a mean FFI increase of 0.99 ± 0.47 units. Both exercise groups significantly improved FFI following 8 weeks of their given intervention (REHIT: *p* < 0.001; MICT: *p* < 0.001). The REHIT group, however, showed improvements in FFI significantly greater than the MICT group (*p* < 0.001).

### Proportion of FFI likely responders and likely non-responders

**Figure 2:**
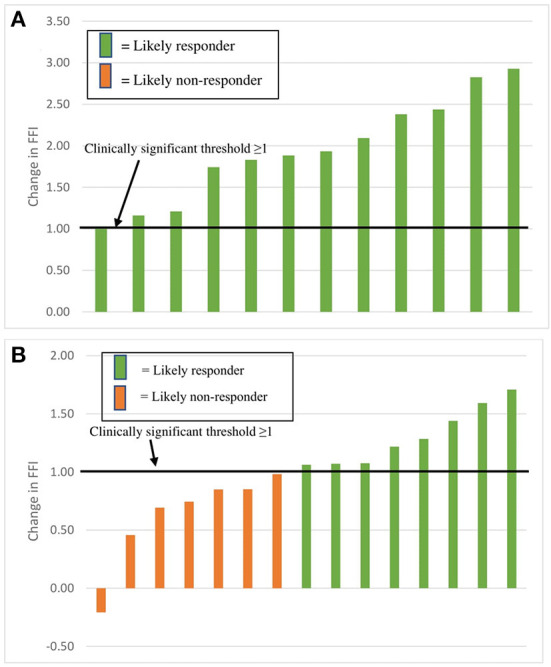
Incidence of likely responders and likely non-responders within REHIT **(A)** and MICT **(B)** groups.

Figure 2 shows the incidence of likely responders and likely non-responders in both the REHIT and MICT groups. The REHIT group had a significantly greater (*p* = 0.01) amount of responders than the MICT group, with 12/12 (100%) participants who performed REHIT improving their FFI by ≥1. The MICT intervention, however, resulted in 8/15 (53%) participants showing an increase of FFI ≥1.

## Discussion

The main finding of this research is that an 8-week REHIT program may be more efficacious at improving FFI relative to a traditional MICT program. The REHIT group showed a mean increase of 1.95 units, compared to a mean 0.99 units increase in the MICT group. Despite total exercise volume throughout the study in the REHIT group being one fifth of that in the MICT group, the REHIT group showed close to 2-fold greater improvements in FFI compared to the MICT group. While both interventions resulted in FFI improvements, there was also a statistically greater proportion of likely responders in the REHIT group. This is evident as 100% of participants in the REHIT group reached the clinically significant improvement of ≥1 unit, while only 53% of those in the MICT group improved their FFI by ≥1. With lack of time being a common barrier to physical activity participation, the outcomes of this research suggest that the prescription of a REHIT program to currently physically inactive individuals would be an efficient and efficacious way of reducing CVD risk.

The outcomes of this research are supported by previous research which found shorter, high-intensity bouts of exercise, similar to REHIT, to be more effective than moderate-intensity exercise prescription (Metcalfe et al., 2012; Ramos et al., 2017; Ruffino et al., 2017). Previously, REHIT has been identified as a more effective modality to improve CRF (Metcalfe et al., 2012; Ruffino et al., 2017), with the improvements in FFI presented in this study further supporting this, as CRF is a component of the FFI. The exact mechanism behind why REHIT is more effective than MICT at improving CRF is still under investigation, given that more volume is usually expected to produce greater improvements in CRF (Thomas et al., 2020). Metcalfe et al. (2015) found significant reductions in muscle glycogen and increased gene expression despite the short duration of REHIT sessions. Furthermore, Metcalfe et al. (2015) found that post exercise oxygen consumption remained higher after REHIT compared to traditional moderate-intensity aerobic exercise. This suggests REHIT may disturb physiological homeostasis to a greater extent in a shorter time, allowing adaptation to occur (Metcalfe et al., 2015). This finding potentially explains the efficacy and efficiency of REHIT compared to traditional methods as seen in the outcomes of this study.

Research has shown that a REHIT intervention has insignificant effect on body composition measures, namely body mass index (BMI) (Metcalfe et al., 2012) and body mass (Ruffino et al., 2017). Similarly, our 8-week REHIT intervention also showed minimal improvements in WtHR, the body composition measure used in the FFI, with the mean WHR in the REHIT group reducing from 0.50 to 0.49. This suggests that improvements in FFI seen in this study may have resulted mostly from an increase in CRF, rather than a change in body composition. While poor body composition is associated with increased CVD risk, CRF is a greater predictor of CVD and all-cause mortality than BMI (Barry et al., 2018), explaining how an increase in FFI may translate to reductions in CVD risk even without significant improvements in body composition.

Despite the efficacy and time efficiency of REHIT, there are still some concerns regarding the prescription of this modality in some populations (Ruffino et al., 2017). One concern relates to the high-intense nature of REHIT. Intensity of exercise is often inversely related to enjoyment (Heinrich et al., 2014), with enjoyment influencing participation and adherence to exercise (Vella et al., 2017). Although there were a greater number of dropouts in the REHIT group, this can be explained through a higher incidence of illness in this group. Additionally, mean adherence was similar in the REHIT group (89.2%) compared to the MICT group (87.8%), despite evidence that intensity is inversely related to enjoyment (Ekkekakis et al., 2011). This could be explained by the length of the intervention however, with evidence supporting the notion that enjoyment of high-intensity exercise programs increases over time through regular participation (Heisz et al., 2016). It is suggested that shorter durations, comparable improvements in strength and fitness, and noticeable increases in workload over time may result in increased enjoyment in high-intensity exercise over time (Heisz et al., 2016). This is further supported by Vella et al. (2017) who found enjoyment in an unsupervised high-intensity exercise program performed was comparable to moderate-intensity aerobic programs in sedentary, overweight and obese individuals. The high adherence rate within this study, as well as evidence suggesting enjoyment in high-intensity exercise increases over time, suggests that REHIT can be prescribed to physically inactive individuals aiming to increase their participation. Further research is required however, to monitor long term adherence to REHIT exercise prescription.

Safety of high-intensity exercise programs such as REHIT has also been identified as a concern when prescribing this type of exercise. As vigorous exercise is associated with a higher risk of an adverse cardiovascular event (Francois and Little, 2015), performing a REHIT training program may similarly increase risk of an adverse event. Despite this, a low risk of the occurrence of acute cardiovascular events when performing other modes of high-intensity interval training has been observed in the Type 2 Diabetes population (Francois and Little, 2015). While there were no adverse events in this study, further research is required to determine the safety of REHIT particularly among other populations.

### Limitations

There are several limitations within this study. Firstly, despite waist circumference being a component of the main outcome measure, diet was not specifically controlled within the study, with participants told to maintain their current diets. Furthermore, physical activity and sedentary behavior outside of the intervention was not monitored. Participants who performed more physical activity other than specified in the intervention may have influenced the results. There was also a greater rate of dropouts in the REHIT group (*n* = 4) compared to the MICT group (*n* = 1). While there was a greater incidence of illness in the REHIT group, this raises questions as to whether the higher intensity REHIT mode of exercise is less tolerable than moderate-intensity exercise. The within-group changes reported here should also be interpreted with caution as we did not have a control group. It is possible that these findings may have been biased by systematic changes over time or with repeated testing. However, this does not affect conclusions with respect to the between-group comparisons between the REHIT and MICT groups. Lastly, it should be noted that our actual group sizes were smaller than our calculated sample size, thus our power size would have been lower than the intended 80% power. Although we were able to detect a difference in the effect between the two exercise interventions, the fact that this was significant despite relatively low statistical power may mean that the effect size was overestimated in the study.

## Conclusion

In conclusion, this research has shown REHIT may be more efficacious than MICT at reducing FFI in physically inactive adults. With lack of time often cited as a reason for not participating in physical activity, this outcome is beneficial as REHIT exercise programs can be performed in as little as 10 min a day. Future research should focus on other populations to determine if REHIT is as effective across populations. Furthermore, long term studies should be performed to monitor adherence to REHIT to determine the efficacy of this mode of exercise as a long-term intervention, to determine whether REHIT is less tolerable than traditional moderate-intensity aerobic training.

## Data availability statement

The original contributions presented in the study are included in the article/supplementary material, further inquiries can be directed to the corresponding author/s.

## Ethics statement

The studies involving human participants were reviewed and approved by Human Research Committee—Western Colorado University. The patients/participants provided their written informed consent to participate in this study.

## Author contributions

Conceptualization, writing–review and editing, supervision, project administration, and resources: JR and LD. Methodology and funding acquisition: LD. Formal analysis: DL, JR, and LD. Writing—original draft preparation: DL. All authors have read, edited, and agreed to the published version of the manuscript.

## Funding

The American Council on Exercise funded this research.

## Conflict of interest

The authors declare that the research was conducted in the absence of any commercial or financial relationships that could be construed as a potential conflict of interest.

## Publisher's note

All claims expressed in this article are solely those of the authors and do not necessarily represent those of their affiliated organizations, or those of the publisher, the editors and the reviewers. Any product that may be evaluated in this article, or claim that may be made by its manufacturer, is not guaranteed or endorsed by the publisher.

## References

[B1] Barry V. W. Caputo J. L. Kang M. (2018). The joint association of fitness and fatness on cardiovascular disease mortality: a meta-analysis. Prog. Cardiovasc. Dis. 61, 136–141. 10.1016/j.pcad.2018.07.004 29981352

[B2] Edwards M. Addoh O. Loprinzi P. (2017). Predictive validity of a fitness fatness index in predicting cardiovascular disease and all-cause mortality. Mayo Clinic Proce. 92:851. 10.1016/j.mayocp.2017.02.013 28473044

[B3] Ekkekakis P. Parfitt G. Petruzzello S. J. (2011). The pleasure and displeasure people feel when they exercise at different intensities. Sports Med. 41, 641–671. 10.2165/11590680-000000000-00000 21780850

[B4] Francois M. E. Little J. P. (2015). Effectiveness and safety of high-intensity interval training in patients with type 2 diabetes. Diabetes Spectrum 28, 39–44. 10.2337/diaspect.28.1.39 25717277 PMC4334091

[B5] Frith E. Loprinzi P. D. (2017). The protective effects of a novel fitness-fatness index on all-cause mortality among adults with cardiovascular disease. Clin Cardiol 40, 469–473. 10.1002/clc.22679 28295468 PMC6490318

[B6] Garber C. E. Blissmer B. Deschenes M. R. Franklin B. A. Lamonte M. J. Lee I.-M.. (2011). Quantity and quality of exercise for developing and maintaining cardiorespiratory, musculoskeletal, and neuromotor fitness in apparently healthy adults: Guidance for prescribing exercise. Med. Sci. Sports Exerc. 43, 1334–1359. 10.1249/MSS.0b013e318213fefb 21694556

[B7] Heinrich K. Patel P. O'Neal J. Heinrich B. (2014). High-intensity compared to moderate-intensity training for exercise initiation, enjoyment, adherence, and intentions: An intervention study. BMC Public Health 14:789. 10.1186/1471-2458-14-789 25086646 PMC4129110

[B8] Heisz J. Tejada M. Paolucci E. Muir C. (2016). Enjoyment for high-intensity interval exercise increases during the first six weeks of training: Implications for promoting exercise adherence in sedentary adults. PLoS ONE 11, E0168534. 10.1371/journal.pone.0168534 27973594 PMC5156428

[B9] Hoare E. Stavreski B. Jennings G. L. Kingwell B. A. (2017). Exploring motivation and barriers to physical activity among active and inactive australian adults. Sports 5, 47. 10.3390/sports5030047 29910407 PMC5968958

[B10] Hussain S. R. Macaluso A. Pearson S. J. (2016). High-intensity interval training versus moderate-intensity continuous training in the prevention/management of cardiovascular disease. Cardiol. Rev. 24, 273–281. 10.1097/CRD.0000000000000124 27548688

[B11] Metcalfe R. S. Babraj J. A. Fawkner S. G. Vollaard N. B. J. (2012). Towards the minimal amount of exercise for improving metabolic health: Beneficial effects of reduced-exertion high-intensity interval training. Eur. J. Appl. Physiol. 112, 2767–2775. 10.1007/s00421-011-2254-z 22124524

[B12] Metcalfe R. S. Koumanov F. Ruffino J. S. Stokes K. A. Holman G. D. Thompson D.. (2015). Physiological and molecular responses to an acute bout of reduced-exertion high-intensity interval training (REHIT). Eur. J. Appl. Physiol. 115, 2321–2334. 10.1007/s00421-015-3217-6 26156806

[B13] Pescatello L.. (2014). ACSM's Guidelines for Exercise Testing and Prescription (9th ed.). Baltimore, MD: Wolters Kluwer/Lippincott Williams and Wilkins Health.

[B14] Ramos J. S. Dalleck L. C. Borrani F. Beetham K. S. Wallen M. P. Mallard A. R.. (2017). Low-volume high-intensity interval training is sufficient to ameliorate the severity of metabolic syndrome. Metab. Syndr. Relat. Disord. 15, 319–328 10.1089/met.2017.0042 28846513

[B15] Ruffino J. Songsorn P. Haggett M. Edmonds D. Robinson A. Thompson D.. (2017). A comparison of the health benefits of reduced-exertion high-intensity interval training (REHIT) and moderate-intensity walking in type 2 diabetes patients. Appl. Physiol. Nutr. Metabo. 42, 202–208. 10.1139/apnm-2016-0497 28121184

[B16] Skinner J. S. Jaskólski A. Jaskólska A. Krasnoff J. Gagnon J. Leon A. S.. (2001). Age, sex, race, initial fitness, and response to training: the HERITAGE Family Study. J. Appl. Physiol. 90, 1770–1776. 10.1152/jappl.2001.90.5.1770 11299267

[B17] Sloan R. A. Haaland B. A. Sawada S. S. Lee I.-M. Sui X. Lee D.. (2016). A Fit-fat index for predicting incident diabetes in apparently healthy men: a prospective cohort study. PLoS ONE 11:E0157703. 10.1371/journal.pone.0157703 27340824 PMC4920380

[B18] Thomas G. Songsorn P. Gorman A. Brackenridge B. Cullen T. Fitzpatrick B.. (2020). Reducing training frequency from 3 or 4 sessions/week to 2 sessions/week does not attenuate improvements in maximal aerobic capacity with reduced-exertion high-intensity interval training (REHIT). Appl. Physiol. Nutr. Metab. 45, 683–685. 10.1139/apnm-2019-0750 32078337

[B19] Vella C. A. Taylor K. Drummer D. (2017). High-intensity interval and moderate-intensity continuous training elicit similar enjoyment and adherence levels in overweight and obese adults. Eur. J. Sport Sci. 17, 1203–1211. 10.1080/17461391.2017.1359679 28792851 PMC6104631

